# Smoking on the margins: a comprehensive analysis of a municipal outdoor smoke-free policy

**DOI:** 10.1186/s12889-016-3466-2

**Published:** 2016-08-22

**Authors:** Ann Pederson, Chizimuzo T. Okoli, Natalie Hemsing, Renée O’Leary, Amanda Wiggins, Wendy Rice, Joan L. Bottorff, Lorraine Greaves

**Affiliations:** 1 BC Women’s Hospital + Health Centre, E305, 4500 Oak Street, Vancouver, BC V6H 3E1 Canada; 2 Faculty of Nursing, University of Kentucky, Lexington, KY USA; 3 BC Centre of Excellence for Women’s Health, Vancouver, BC Canada; 4 University of Victoria, Victoria, BC Canada; 5 University of Kentucky, Lexington, KY USA; 6 Institute for Healthy Living and Chronic Disease Prevention, University of British Columbia, Kelowna, BC Canada; 7 Faculty of Health Sciences, Australian Catholic University, Melbourne, Australia

**Keywords:** Tobacco control, Health equity, Outdoor smoking ban, Marginalization, Municipal, Policy, population health intervention, Park, Beach, Canada

## Abstract

**Background:**

This study examined the formulation, adoption, and implementation of a ban on smoking in the parks and beaches in Vancouver, Canada.

**Methods:**

Informed by Critical Multiplism, we explored the policy adoption process, support for and compliance with a local bylaw prohibiting smoking in parks and on beaches, experiences with enforcement, and potential health equity issues through a series of qualitative and quantitative studies.

**Results:**

Findings suggest that there was unanimous support for the introduction of the bylaw among policy makers, as well as a high degree of positive public support. We observed that smoking initially declined following the ban’s implementation, but that smoking practices vary in parks by location. We also found evidence of different levels of enforcement and compliance between settings, and between different populations of park and beach users.

**Conclusions:**

Overall success with the implementation of the bylaw is tempered by potential increases in health inequities because of variable enforcement of the ban; greatest levels of smoking appear to continue to occur in the least advantaged areas of the city. Jurisdictions developing such policies need to consider how to allocate sufficient resources to enhance voluntary compliance and ensure that such bylaws do not contribute to health inequities.

## Background

Smoke-free policies are a valuable population health intervention to address one of the most common and significant global threats to health [1]. When effectively implemented and adequately enforced, smoke-free policies targeting public spaces have been instrumental in reducing smoking and improving health outcomes at the population level [2]. However, this evidence has primarily come from policies that address smoking in indoor and adjacent outdoor public spaces. Few studies have examined the effectiveness of such policies targeted towards outdoor recreational spaces, such as parks and/or beaches [3, 4].

Controversy surrounding the extension of smoke-free policies into outdoor spaces [5–7] has been minimized by recent strong evidence of the substantial tobacco smoke exposure that may occur in outdoor spaces [8–11]. Arguments for the adoption of outdoor smoke-free policies often focus on reducing the detrimental health effects of secondhand tobacco smoke (SHS) exposure (given that there is no-known ‘risk free’ level of such exposures), the denormalization of smoking, aesthetic issues related to cigarette litter, and safety concerns related to fires that can arise from cigarette smoking in high risk areas [12]. Another related and often highlighted reason for adopting smoke-free policies is an emphasis on the need to protect nonsmokers, especially children, from tobacco smoke exposure in public places [3, 13]. Public support for such policies is increasing and offers municipal officials a rationale for their adoption [3]. To date, few studies have examined the implementation and enforcement of outdoor smoking bans, focusing instead on the policy development and adoption process though the small number of studies which have addressed enforcement of outdoor smoke-free policies suggest that potential enforcement issues are more of a concern in jurisdictions that have not adopted a policy but that few problems occur in jurisdictions that have adopted such policies [14]. Our research suggests, however, that enforcement and compliance remain concerns and warrant further study.

Moreover, while there is increasing use of smoke-free bylaws in parks and on beaches (as of January 2016, the Non-Smokers’ Rights Association reported that over 52 municipalities now have restrictions of smoking in beaches and 85 prohibit smoking in parks in Canada (see http://database.nonsmokersrights.ca)), there has been relatively little examination of their effects, especially with respect to health equity. In 2008, the WHO Commission on the Social Determinants of Health recommended that reducing health inequities—that is, avoidable health inequalities—be considered a goal of health and social policies [15]—which should include tobacco control. However, as a tool for health equity [16, 17] smoke-free policies may not be equally effective among all populations of smokers or all settings, and may even contribute to exacerbating existing health and social inequities, depending upon the effectiveness of their implementation [18]. In a review of population tobacco control interventions and their effects on social inequalities in smoking, Main et al. [19], for example, found that few systematic reviews attempted to examine differential intervention impacts between population groups. They concluded that while there is clear evidence of the effectiveness of some tobacco control interventions in reducing overall population smoking prevalence, the health benefits are not equitably distributed [19].

### Background

In 2013, smoking prevalence in the Vancouver Health Authority, which includes the City of Vancouver, was 15.9 % for all smokers (daily and occasional combined), which is similar to the provincial (British Columbia) rate of 16.2 % [20, 21]. The smoking prevalence for non-Canadian born residents is lower than for native born Canadians, 5.4 % compared to 14.7 % (possibly arising from the healthy immigrant effect or cultural differences [22] because of a higher proportion of immigrants in Vancouver relative to some other cities in Canada [23]. There is a gender difference in prevalence: 21.3 % for males and 10.8 % for females. Rates of SHS exposure in vehicles and other public spaces in the previous month (among both smokers and nonsmokers) were 15.2 % for males and 14.0 % for females. In British Columbia, there are higher rates of smoking among groups who are vulnerable to disadvantage such as those on low-income and those of aboriginal ethnicity. Smoking rates are inversely correlated with household income: 17.4 % for under $20,000; 12.5 % for $20,000–$39,999; 10.4 % for $40,000–$59,000; 7.4 % for $60,000–$79,000; and 6.3 % for $80,000+. No survey data were available for Aboriginal prevalence rates in Vancouver in this dataset. Age-standardized Statistics Canada data for those with Aboriginal status in British Columbia for 2007 to 2010 estimated 31.6 % overall smoking prevalence (29.7 % for males and 33.1 % for females) and among non-smokers SHS exposure in the home was 8.7 % (7.7 % among males and 9.6 % among females) and SHS exposure from a vehicle or public place in the past month was 22.2 % (25.3 % for males and 19.5 % for females) [24].

**Fig. 1:**
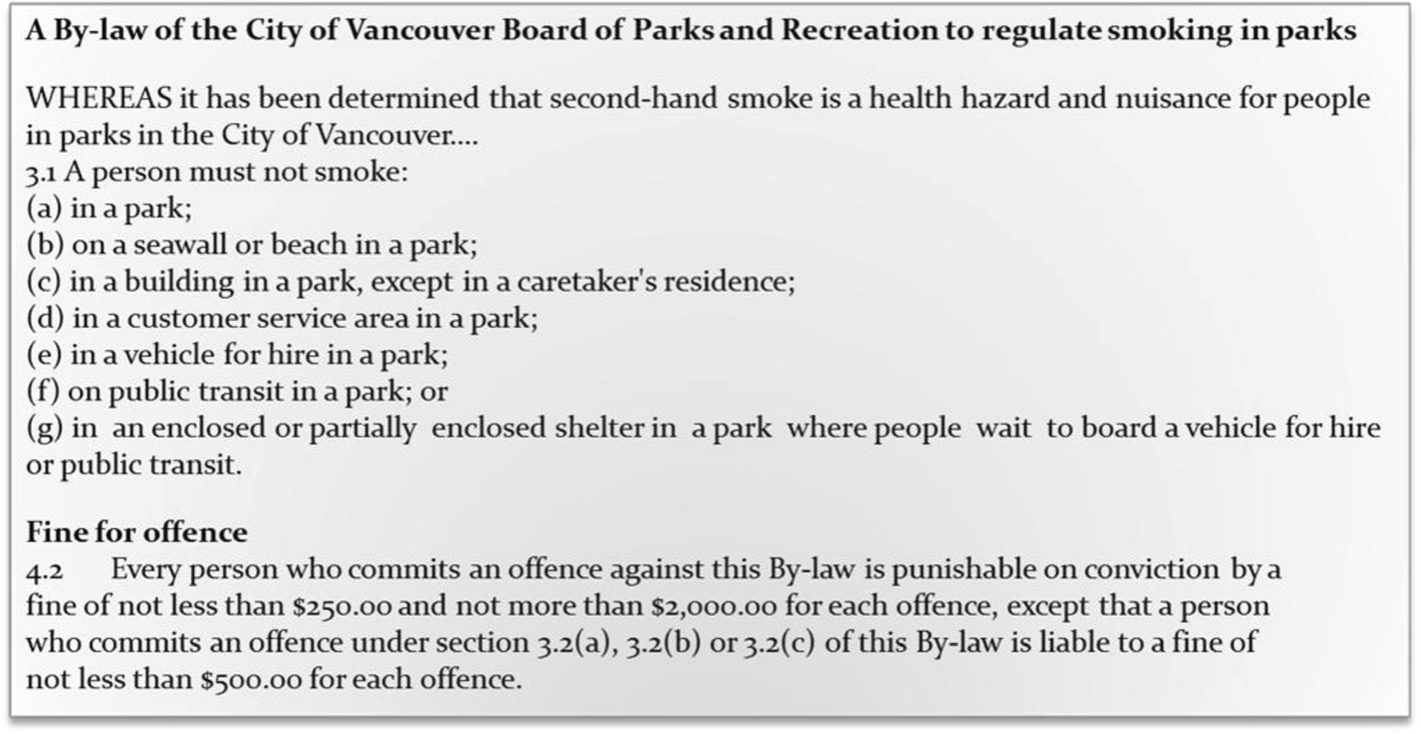
City of Vancouver Bylaw

Source: Extracted from *A By-law of the City of Vancouver Board of Parks and Recreation to regulate smoking in parks*, Appendix A (#131148v8). Retrieved September 5, 2011 from: http://vancouver.ca/files/cov/park-smoking-regulation-bylaw.pdf.

British Columbia initiated smoking restrictions in 1984 by requiring non-smoking areas in retail establishments, restaurants, and bars. In 1999, smoking was banned completely in all indoor public spaces, based on the evidence of the health effects of second hand smoke. In Vancouver, the most populous city in British Columbia, City Council banned smoking within six meters of any door, window, or air intake, and also on outdoor patios of bars and restaurants in 2007. In keeping with this history of strong tobacco control efforts, a bylaw prohibiting smoking in parks and on beaches was approved by the City’s Board of Parks and Recreation on June 22^nd^, 2010 and came into effect September 1^st^, 2010. This bylaw, which is limited to the geographic area governed by the municipal government and its elected Park Board, prohibits the smoking of any substances (including e-cigarettes, marijuana, and combustible tobacco in any form) in Vancouver’s parks, beaches, and recreational facilities (see Fig. 1).

The purpose of this study was to critically assess the adoption and implementation (including compliance and enforcement) of a ban on smoking in the parks and beaches in Vancouver, Canada. Four overarching research questions guided our study as follows:

1. What was the process of adoption of the bylaw?
2. To what extent is the bylaw being supported and adhered to?
3. What is the process of enforcement?
4. What are the health equity impacts of the bylaw?

## Methods

We employed a mixed methods approach to evaluate the policy *in context* as has been performed recently in other studies examining bylaw restrictions in New York City [25]. We adopted Critical Multiplism [26] as the methodological frame for the study, which acknowledges that different research methods contain weaknesses and biases which necessitate the use of multiple methodological approaches to minimize the influence of any single bias. Accordingly, we use the findings from nine different sources of evidence (see Table 1) to generate an assessment of the bylaw by examining policy adoption, support and compliance, and enforcement. We particularly wanted to understand the processes of policy adoption, support, compliance, and enforcement from the perspective of health equity. In 1992, Margaret Whitehead [27] defined health inequities as “differences health that are unnecessary, avoidable, unfair and unjust”, while Braveman and Gruskin [28] have argued that,

“…equity in health is the absence of systematic disparities in health (or in the major social determinants of health) between groups with different levels of underlying social advantage/disadvantage—that is, wealth, power, or prestige. Inequities in health systematically put groups of people who are already socially disadvantaged (for example, by virtue of being poor, female, and/or members of a disenfranchised racial, ethnic, or religious group) at further disadvantage with respect to their health.”

**Table 1:** Description of SOTM studies

Study name	Research question	Design and Methods
Document review	Adoption	Design: Review of online, official Park Board meeting records, City Council minutes and commentaries, and the results of the Park Board’s pre-law public opinion survey from 2007–2012. Analysis strategy: Documents were analyzed thematically to understand the reasons for adopting the bylaw.
Key informant interviews	Adoption	Design: Semi-structured interviews. Sample: Eight key informant interviews with civic officials, public health advocates and health care providers conducted from May to December 2011. Analysis strategy: Recorded interviews were transcribed verbatim, and analyzed thematically to generate a chronological account of the introduction of the bylaw and to understand the reasons the informants had for supporting or opposing the bylaw.
Social and built environment study [59]	Support	Design: Semi structured interviews and focus groups between March 2010 and February 2011 (prior to the implementation of the smoke-free bylaw) Sample: 40 telephone interviews (with 21 women and 19 men in Greater Vancouver) and focus groups with seven additional participants who were exposed to secondhand smoke daily or almost daily. Analysis: Recorded interviews were transcribed verbatim and analyzed thematically to obtain information on support for the bylaw.
Media analysis [42]	Support	Design: Content analysis of print news media from January 1, 2010 to December 31, 2011. Sample: 90 articles from the Canadian Newsstand Database and independent newspapers. Analysis: Articles were coded in two stages, first using a custom Perl script and then with a set of content variables. The articles were further coded using 45 content variables into the categories of relevance, geographic focus, slant, primary approach, theme, and tobacco control topics.
Park User Telephone Survey [43]	Support	Design: A cross-sectional survey using a random digitalized calling sampling process between September 15^th^ and 25^th^, 2011. Sample: 496 Vancouver residents (446 nonsmokers and 50 smokers) who had visited a beach or park in the previous year (from Sept 2010 to Sept 2011) —the first year of the smoking bylaw. Analysis: Data obtained from respondents included demographic information, smoking status, support for the smoke-free bylaw, and opinions regarding the smoke-free bylaw. Unadjusted and adjusted logistic regression analyses were used to examine the correlates of supporting the bylaw.
Park and Beach Observation Study [60]	Compliance	Design: Observations of parks and beaches at nine time-points (pre-bylaw, one-week, one-month, 8-months, 9-months, 10-months, 12-months, 22-months, and 24-months after bylaw implementation) from August 2010 to September 2012. Sample: Purposively selected parks (*n* = 3) and beaches (*n* = 3) in Vancouver, Canada. Analysis: Observed smoking in each venue was recorded during a 30-min time period. Observation sessions were limited to afternoons and evenings on the weekends (Friday-Sunday). Information on the maximum number of persons, total number of smokers, duration of time spent, and average daily temperature were recorded per venue. Friedman’s tests were used to assess the changes in the total smoking rates in venues over time. Wilcoxon signed rank tests were used to assess the differences between prelaw and each subsequent observation time point smoking rates. Mann–Whitney tests were used to examine the differences in smoking rates between parks and beaches.
Beach Litter Study	Compliance	Design: Secondary analysis of observational data from the Great Canadian Shoreline Cleanup (see http://www.shorelinecleanup.ca/) which comprised park and beach litter data from one year before and two years after the implementation of the bylaw from 2010**–**2012. Sample: Litter (from cigarettes/cigarette filters, tobacco packaging, cigarette lighters, and cigar tips) among 40 sites from which litter was consistently obtained in all 3 data collection periods. Analysis: For each venue, information on number of volunteers, distance cleaned, and litter (cigarettes/cigarette filters, cigarette lighters, cigar tips, tobacco packaging) was obtained. Repeated measures analysis for negative binomial regression was based on the generalized estimating equation (GEE) approach and was used to evaluate differences in the amount of litter obtained between parks and beaches over the 3-year study period. Each model included the factors of venue type (park vs. beach), year (2010, 2011 and 2012) and the interaction between venue type and year as well as the time-dependent covariates for number of volunteers and kilometers covered.
Park Ranger Focus Group and Citation Information	Enforcement	Design: Two semi-structured focus groups in October 2011 (13 months following implementation) and then again a year later in August 2012. Citation data was obtained from the metro police department. Sample: Twelve individuals participated in the focus groups (6 individuals participated in both groups). Rangers who participated in the focus groups included novices and senior officers (8+ years), and both seasonal and permanent employees. (The permanent Park Ranger contingent is tiny, consisting of a full-time Lead Ranger, a full-time Homeless Liaison, and four part-time Rangers; in the summer months, when park and beach usage peaks, 36 seasonal auxiliary Rangers join the permanent staff). Analysis: Focus group data were analyzed with a 23-item coding frame created by the Principal Investigator (PI) and a team member. Inter-coder reliability with a third team member was .849 (Krippendorff's alpha). A saturation of themes was demonstrated when no additional codes were created during the coding process. A narrative summary was compiled for each code for each focus group, and the number and density of responses analyzed; the two focus groups were also compared for changes from year one to year two. The identification of themes was formulated by the PI in conjunction with the research team. Citation data from Municipal Ticket Information system reported as frequencies.

In the context of tobacco control, Ritchie, Amos & Martin [29] have argued that “Smoking is a major cause of inequalities in health in many high income countries” (p. 461). A recent U.S. study found that lower SES communities were less likely to adopt outdoor smoke-free laws as compared to higher SES communities [30]. Several studies have further established that lower socioeconomic status (SES) is significantly predicts poor smoking cessation [31]. For example, a recent study from the UK among 3057 clients of smoking cessation services found that those with higher SES were 1.4 (95 % CI = 1.1-1.9) times more likely to achieve cessation as compared to those at a lower SES [32]. A longitudinal study in 11 European countries found that although overall smoking cessation rates increased for both low and high SES groups as a result of tobacco control policies during 1987–2012, the cessation ratio between the two groups also significantly increased [33]. These findings suggest that tobacco control policies that were implemented during the 2000’s in those countries did not mitigate socioeconomic inequalities in smoking cessation [33]. In a similar fashion, SES differences have been consistently demonstrated in SHS exposure [34]. However, the health inequalities related to the association between SHS exposure and SES has not been adequately examined in Canada. A recent study, however, identified potential subpopulations at greater risk of SHS exposure in Canada, such as children, adolescents, and those exposed to SHS in the home environments [35]. Hence, addressing potential social and health inequalities of tobacco policies in Canada is crucial given the evidence that some tobacco control initiatives (such as workplace interventions and tobacco pricing) may exacerbate inequalities among those with different SES levels [36].

In the present study, we were concerned with two aspects of Mahoney et al.’s [37] *Equity-focused Health Impact Assessment Framework* (EFHIA), namely, a) any differential impacts of the smoking ban across the population and b) what, if any, measures were used to balance the burdens/benefits of the policy across the population. Hence, we considered such equity issues using Mahoney et al.’s approach to equity-focused health impact assessment [27, 37] and reviewed debates in the tobacco control literature [5, 29, 38–40] to inform our critical analyses of policy adoption, support, compliance, and enforcement. The protocols for all studies were approved by the UBC/Children’s & Women’s Health Centre Research Ethics Board (Certificate Number: CW19-0185/H10-01801).

## Results

### Policy adoption

To examine the policy adoption process, we critically reviewed available public documents on the bylaw and narrative data from key informants regarding the process of policy adoption.

#### Document reviews and key informant interviews

**Fig. 2:**
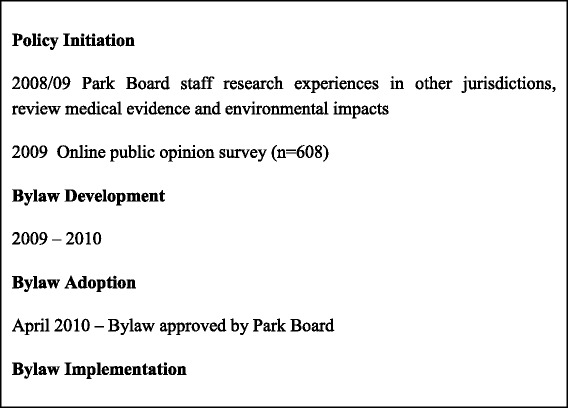
Milestones in the Development of the Outdoor Smoke-free Policy in Vancouver

From the document review and key informant interviews, it was clear that there was both official and public support for the introduction of the bylaw (see Fig. 2). In fact, the recommendation to introduce the bylaw generated only limited discussion at the Park Board session at which it was debated and most of the discussion focused on health concerns. One Park Board councillor even questioned whether there was any need to hold a discussion prior to voting for the bylaw, presumably because it was felt that the case for supporting it was so transparently sound. To the extent that they were evident at all, equity issues were discussed with respect to whether non-smokers, especially children, had the right to be free of smoke in outdoor places. The Park Board and City Council (which had to be involved for legal reasons) both agreed that the enforcement would be handled by Park Rangers (and police) though there was hope that the bylaw would be self-enforcing through signage and social pressure. To enhance public/self-enforcement, an educational intervention was started prior to the bylaw coming into effect to raise awareness of the ban. The majority of the key informants, all of whom were familiar with the process of bylaw development in Vancouver, confirmed that equity issues were not part of the debate or decision-making process, and though they were generally supportive of the bylaw as a public health intervention, some questioned its implications for civil liberties and fairness.

### Public support and compliance

To assess support for and compliance with the bylaw, we conducted a series of studies employing qualitative and quantitative approaches to using narrative, media, survey, and observational data.

#### Social and built environment study

At the time the smoke-free bylaw was announced, some of our research team were collecting data regarding SHS exposure in Vancouver [41]. A convenience sample of 47 low-income and non-low-income men and women of varied smoking statuses was recruited to participate in a telephone interview or a focus group. A subset of these study participants (eight individuals who completed one-on-one individuals and one focus group of four), spontaneously remarked on the new bylaw in the city and these results are discussed here.

The majority of study participants who commented on the proposed smoking ban on beaches and in parks disagreed with the implementation of the ban. Reasons for opposition included: concerns over infringement on the rights of smokers, the potential for stigma, and issues with enforcement and compliance. For example, one participant noted:

“I think in public places if people want to rest and stop and have a social interaction while they’re having a cigarette then there needs to be designated smoking areas because that’s the – I think an outright ban of people on beaches is just not the way to go” (female non-smoker, April 19, 2010).

Several participants commented that smokers are capable of managing their smoking in outdoor spaces and respecting the rights of nearby non-smokers. To support their stance, they suggested that such bans would be indicative of a “nanny-state” and defended the legality of smoking and argued that there were practical challenges with enforcement.

While most participants who spoke about smoking bans on beaches and parks were critical or concerned about effectiveness, two non-smoking participants (one female, one male) expressed complete support for public smoking bans. One participant, who had been lobbying the city to implement smoking bans on beaches and parks, commented:

“So I’m hoping that they’re going to pass this [ban on smoking in beaches and parks] and put it through to clean up the air for most of us. That would be wonderful, a dream come true” (male, non-smoker, April 16, 2010).

Reasons for supporting the ban included a reduction in cigarette-related litter, increase in smoking reduction and cessation with expanding denormalization of smoking, a reduction in children’s exposure to SHS, and the discomfort engendered by SHS exposure (one participant noted that he could smell someone smoking “two blocks away”).

In sum, there was variation in both tolerance for SHS and support for smoking restrictions among this study’s interviewees. The results of this study offered a window into the range of opinions and experiences of both women and men, smokers and non-smokers, and help set the context for our other data collection processes and analyses.

#### Media analysis

We examined newspaper coverage of the smoking ban prior to and following implementation. Articles were separated into three categories: news stories (60 %, *n* = 54), letters to the editor (18.9 %, *n* = 17), and opinions and editorials (21.1 %, *n* = 19). We observed the greatest newspaper coverage when the bylaw was announced in April 2010, with a total of 19 articles published, although a small number of articles were published every month during the study period. The April 2010 announcement also had the largest number of letters to the editor (*n* = 9).

An analysis of article slant showed differences in views towards the bylaw: for the news articles, 50 % (*n* = 27) were positive and 7.4 % (*n* = 4) negative, while for the letters to the editor only 23.5 % (*n* = 4) were positive and 64.7 % (*n* = 11) were negative. The most frequent topic related to enforcement and implementation (i.e., signage, enforcement officers, and implementation issues: 64 articles), followed by unintended consequences of smoking (i.e., litter, fire, public nuisance: 39 articles), and second hand smoke exposure (31 articles). Equity issues (i.e., the rights of smokers and non-smokers, fairness of the law) was discussed in only 21 articles [42].

#### Park user telephone survey

The bylaw was supported by 85 % (*n* = 421) of survey respondents with a significantly greater proportion of females supporting the bylaw than males (89 % vs. 78 %), and a significantly greater proportion of nonsmokers supporting the law than smokers (89 % vs. 52 %). Beliefs regarding the bylaw were that it would: improve the health of people in the city (total = 82 %, nonsmokers= 86 % vs. smokers = 43 %); protect the health of non-smokers, including children who visit parks and beaches (total = 83 %, nonsmokers = 86 % vs. smokers = 56 %); encourage people to quit smoking (Total = 49 %, nonsmokers = 52 % vs. smokers = 22 %); discourage youth from starting smoking (Total = 49 %, nonsmokers = 50 % vs. smokers = 33 %), infringe on the right of smokers (total = 42 %, nonsmokers = 39 % vs. smokers = 71 %); and protect people from exposure to secondhand smoke (total = 84 %, nonsmokers = 88 % vs. smokers = 52 %) [43]. Women were significantly more likely than men to believe that the bylaw would protect the health of nonsmokers (including children) who visit parks and beaches (64.1 % vs. 35.9 %) and protect people from exposure to secondhand smoke (63.6 % vs. 36.4 %). There were no further significant gender differences in beliefs regarding the bylaw.

Results of multivariate analysis suggest that favourable beliefs regarding the bylaw were associated with increased support for the law. Support for the bylaw varied by sex, self-identified ethnicity, education and marital status. Females were significantly more likely to support the bylaw than were males (aOR = 2.8, 95 % CI = 1.5-5.1); individuals from different visible minority groups were significantly more likely to support the law than those from White or European Ancestry (aOR = 2.1, 95 % CI = 1.0-5.0); and those with a university degree were significantly more likely to support the law as compared to those with a high school degree or lower (aOR = 2.5, 95 % CI = 1.1-5.5) [43]. Never married individuals were significantly less likely to support the law than those who were married (aOR = 0.5, 95 % CI = 0.2-1.0) [43].

Although the majority of residents participating in the telephone survey endorsed the smoke-free bylaw, they recognized its potential for stigmatizing smokers and smoking. About three-quarters agreed that the bylaw could increase negative attitudes or stigma towards smokers. Smokers were significantly more likely to voice concerns that the bylaw infringes smokers’ rights (71 % vs. 39 %) [43]. Thus survey respondents, particularly if they were smokers, recognized potential negative consequences.

#### Park and beach observational study

**Fig. 3:**
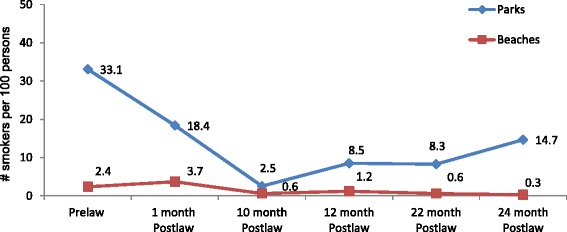
Changes in Mean Smoking Rates in Selected Parks and Beaches (Pre-bylaw to 24-month Post-bylaw)

A total of 23,815 persons were observed in selected parks and/or beaches during the observation time points from 2010–2012 with a median of 11.5 smokers (min = 0.0 to max = 32.0) and a median smoking rate of 4.8 smokers per 100 persons (min = 0.0 to max = 64). Parks had significantly higher smoking rates as compared to beaches (mean rate parks = 17.9 vs. beaches =1.9). Significant changes in smoking rates were observed overall from pre-bylaw to 24-months post-bylaw (pre-bylaw mean rate = 20.6 vs. 24-month mean rate = 8.6). In stratified analyses (see Fig. 3), changes in mean smoking rates were significant in both beaches and parks. However, the differences between pre-bylaw, 12-month, and 24-month were no longer significant in the stratified analyses.

Despite the inherent weaknesses in design (such as lack of randomization of observed venues), these findings are strengthened by multiple detailed observations carried out in the same venues. Total observed smoking rates in all venues decreased over time; however, no venue had 100 % compliance with the smoke-free bylaw. Moreover there was lower compliance in the parks as compared to the beaches.

#### Beach litter study

**Fig. 4:**
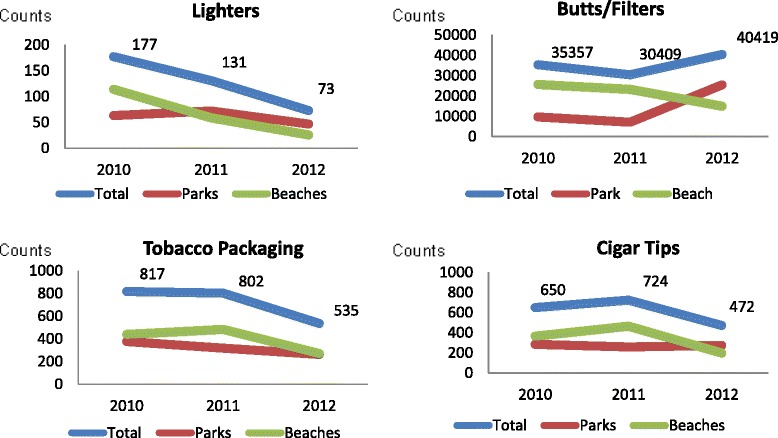
Beach litter data 2010–2012

The number of lighters, cigarette butts/filters, cigar tips and/or packaging found were analysed by venue type, year, number of volunteers and/or distance covered (Fig. 4). The following significant relationships were found: there was a decline in the number of lighters obtained, with significant reductions between years 2010 and 2012 (*p* = .005) and years 2011 and 2012 (*p* = .016). Cigarette butts/filters were more likely to be obtained from beaches than parks (RR = 1.98 (95 % CI 1.13, 3.48).

Our analysis of the park and beach litter data suggest that in 2010 there were more cigarette/cigarette filters on beaches (*n* = 25687) than parks (*n* = 9670), but by 2012 there were a larger number of cigarette/cigarette filters in parks (*n* = 25456) than on beaches (*n* = 14963). However, the change in total beach litter counts was not significant (with the exception of cigarette lighters) over the three year data collection time point (see Fig. 4).

### Enforcement

To assess the enforcement of the bylaw, we collected narrative data from enforcement officers through focus groups and obtained information on issued citations due to violations of the bylaw.

#### Park ranger focus groups and citation information

As reported by key informants and in Park Board minutes, Vancouver opted to have its small contingent of Park Rangers function as bylaw enforcement officers as part of its implementation strategy. Although members of the Vancouver Police Department were also given the authority to enforce the ban, bylaw enforcement per se was not a new function for them nor were they expected to be the main enforcers of this bylaw, so they were not interviewed for this study.

Though it is not the focus of the present discussion, it is noteworthy that the introduction of the smoking ban represented a major change in the work of the city’s Park Rangers. Prior to the introduction of the bylaw, the Rangers regarded themselves as “ambassadors” for the parks. Following the introduction of the ban, they had to learn how to function as “bylaw enforcement officers”, a more policing role. During the focus groups, the Park Rangers described various aspects of their day-to-day enforcement experience. Among their observations was the fact that they witnessed and participated in differential enforcement based on locations and populations.

Given available staffing, the Rangers reported that there was consensus among management that it would be impossible to cover all the city’s parks and beaches, so popular tourist beaches were subject to the most enforcement. At the same time, Rangers explained that ticketing was not done in specific parks "for safety reasons" because some parks located in the city's lower-income areas were "charged environments", that is, environments in which visitors were more resistant to enforcement and could be threatening to the enforcement Rangers.

According to the Rangers, some populations were not subject to enforcement. For example, we learned that Park Board Department policies exempt the homeless and tourists from ticketing. Uncooperative violators could also avoid tickets by refusing to supply identification; indeed, Park Rangers do not have the authority to demand identification. The Rangers observed that these “scoff-laws” were often from the less affluent East Side of the city. Rangers also explained that First Nations individuals would sometimes protest that the bylaw did not apply to them given land claim and jurisdictional disputes, and that this put the Park Rangers into "a weird position". Other Rangers pointed to the general policy of Ranger discretion, which meant that they could withdraw from potential enforcement actions with uncooperative violators. Finally, some Rangers said they targeted cannabis users for enforcement rather than tobacco smokers, rationalizing that cannabis use is (still) illegal in Canada independently of this particular bylaw and hence they found it easier to justify its enforcement.

**Fig. 5:**
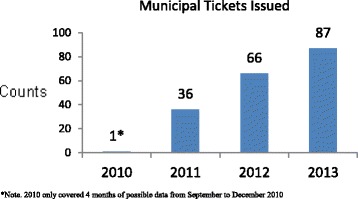
Citations issued by year since implementation of bylaw 2010–2013

Few Rangers acknowledged the possible consequences of differential enforcement. Some Park Rangers explained they were initially reluctant to enforce the bylaw, particularly in the parks, whereas there was greater confidence about both the legitimacy of enforcing the bylaw over time and about the process for doing so (following specific training). This is supported by data indicating that initially warnings were the primary form of ticketing following the approval of the smoke-free bylaw, but the use of fines increased with time (see Fig. 5).

Some Park Rangers commented that enforcement is likely to have different consequences depending upon the circumstances of the person of interest. For example, a bylaw infraction could result in a $250 fine; this expense for noncompliance burdens low-income smokers more than more affluent smokers. Yet without robust enforcement in parks in the lower-income areas, lower-income populations and marginalized communities could also receive less protection from SHS, and derive less tobacco control benefit (denormalization, temporary abstinence, quit motivation). These populations are already at greater risk for tobacco use and exposure. In the early phase of implementing the ban, the Park Rangers noted that enforcement focused on education and warnings; as noted, ticketing for non-compliance increased with time (see Fig. 5) even as these other aspects of enforcement continued.

## Discussion

This study is one of the first to conduct a comprehensive, multi-year assessment of the adoption and implementation of a municipal bylaw prohibiting smoking in parks and on beaches. The use of critical multiplism to organize the study proved to be a useful approach to understanding a complex population health intervention in context as it explicitly involved collecting, comparing and reflecting upon multiple forms and sources of data over time. The various methods generated complementary findings regarding the policy-making process and the nuances of implementing an outdoor smoke-free policy. For example, despite evidence of some opposition and resistance to the introduction of the outdoor smoke-free bylaw in Vancouver from civil rights groups and smokers, the public documentary record, our telephone survey, the newspaper media analysis, and key informant interview data confirm a high level of support for the bylaw. This degree of support is consistent with studies in other jurisdictions, including Britain, New Zealand, Australia, Canada, and the United States [44, 45], and should be encouraging to policy makers and public health advocates.

A closer examination of the literature on public support for smoke-free bylaws indicates that proponents support such policies because they denormalize smoking and limit children’s exposure to smokers and tobacco smoke [3, 44]. For example, a recent Canadian study found very strong parental and caregiver support for smoke-free polices in playgrounds to reduce potential health risks associated with SHS exposure among children [13]. However, our park user telephone survey suggested that park users in Vancouver differed regarding support for the bylaw by smoking status, gender, ethno-cultural affiliation, education, and marital status [43]. That is, smokers were less supportive of the bylaw; women were significantly more likely to support the bylaw; individuals from various visible minority groups were significantly more likely to support the law than those of White or European Ancestry; and those with a university degree were significantly more likely to support the law as compared to those with a high school degree or lower. Findings from our media analysis suggested that support for the bylaw was captured in different forms (i.e., letters versus news items) in the print media. Other media analyses also suggest that the extent and nature of news coverage is associated with variation in support for smoke-free bylaws [46]. These findings are consistent with other studies of the introduction of smoke-free laws [47] and suggest that implementers should be mindful of both opportunities to mobilize support and address resistance.

We also uncovered skepticism about the likely effectiveness of the ban, the risks of SHS in an outdoor setting, and awareness that such bylaws increase the stigmatizing of smokers and smoking. Similar concerns have recently been documented in other studies [38, 44, 48]. Our focus groups and interviews with smokers and nonsmokers and findings from the public opinion survey, suggest that a number of respondents expected the bylaw to increase the stigmatization of smokers. This challenge of greater stigmatization and its potential consequences is a concern in current tobacco control efforts (e.g., [38, 49]). Increased feelings of being stereotyped and stigmatized have been reported by smokers facing increasing restrictions on public smoking in other studies [48]. Such smoking restrictions, which increase the social unacceptability of smoking, may further marginalize these groups and create more barriers to accessing health services [38, 50, 51]. Further, these processes are gendered, in that women and men who smoke may face different degrees of stigma [52] and differential access to private and public recreation spaces [53, 54]. Based on these findings, it may be important to include sub-groups (such as male and female smokers and low-income individuals) when developing implementation plans for outdoor smoke-free policies to potentially mitigate unintended adverse effects of such policies.

Overall lower rates of compliance with the smoke-free law were observed in our study in the parks which were located in lower-income areas of Vancouver relative to beaches, which are primarily located in the more affluent areas of the city. This finding can be explained in part by the higher smoking prevalence in the lower-income areas and the lack of prioritization of bylaw enforcement by Park Rangers in these areas. These findings are consistent with the results of the study reported by Ritchie et al. [29] which demonstrated that post-regulation use of public spaces was related to a variety of pre-legislation differences in the communities and the ways that people engaged in particular social and cultural spaces. Similar patterns have also been observed in a recent study that found improvements in smoking behaviours (e.g., decreased cigarette consumption and increased quitting) among affluent localities but little improvements in less affluent localities after the introduction of a smoke-free law in Scotland [29]. However, our finding of a minimal reduction in smoking-related litter is somewhat different from the results of other studies, such as the one conducted by Johns et al. in New York City [55]. The lack of significant change in the volume of cigarette-related litter in Vancouver suggests the need for greater enforcement of this aspect of the smoke-free bylaw.

An important element of this project was assessing whether equity concerns were an element of policy development and implementation. The historical document trail, key informant interviews and media analysis suggested there was almost no concern expressed in the policy adoption phase with issues of fairness or differential effects. However, important equity concerns were raised during discussions with the Park Rangers regarding their experiences of actual, day-to-day implementation of the smokefree policy. For example, they acknowledged differential enforcement levels at various sites (more enforcement at beaches than parks, for example). We also learned that some groups of park users are exempt from ticketing while others are subject to the discretion of the enforcement officers. Yet without adequate enforcement, park visitors in lower-income areas of Vancouver may continue to disproportionately experience SHS exposure while visitors in the parks and on the beaches of wealthier neighbourhoods are more likely to be protected.

By applying an equity lens to all our findings, a fine tension emerges between the potential unintended consequences of the bylaw (i.e., stigmatizing an already disadvantaged groups of smokers with low socioeconomic status) and the detrimental effects of inadequate enforcement and non-compliance (i.e., increased risk of the same disadvantaged groups to the adverse health effects of SHS exposure). There may be no direct solution to this conundrum. For example, designated smoking areas in outdoor venues, have been shown to continue being a source of significant SHS exposure [56]. Creative approaches to enforcement of the bylaw may mitigate the degree of the inequitable effects of the policy. For example, those who enforce the bylaw may provide tobacco treatment assistance (i.e., brochures, vouchers for medications) and other similar resources to violators of the bylaw; or repeat offenders may be asked to attend mandatory tobacco treatment in lieu of fines. Nonetheless, for the smoke-free policy to be maintained as intended, it will be necessary to have ongoing bylaw communication and investments in resources for enforcement.

Our equity analysis was informed by tools such as Mahoney et al.’s EFHIA [37], which encourages questioning throughout all phases of a project, including a potential policy, not simply applying an equity analysis to intervention outcomes. The aim of EFHIA is to “put equity and health on [the] agenda in a more obvious and systematic way” (p. 1) and was therefore a useful starting point for generating questions for our data collection processes regarding the extent to which equity concerns were or were not in the minds of key decision makers throughout the policy development, adoption and implementation process. Critically, EFHIA asks whether any observed difference in health outcome or its precursors—in this case, for example, exposure to SHS—is avoidable and unfair. Although health equity has had significant international attention since the publication of the WHO Commission on the Determinants of Health report in 2008 [15], it is clear that additional tools, strategies and supports for applying an equity lens are still needed, including in the field of tobacco control, particularly with regard to smoking prevention or cessation supports for disadvantaged groups of smokers. As Beauchamp et al. [57] suggest, underlying material, social and environmental factors associated with disadvantage are likely to present significant barriers to the effectiveness of interventions.

This study has several limitations that need to be considered in interpreting the findings. This study employed a mixed-methods approach, employing both qualitative and quantitative approaches to data collection and analysis. The findings from our qualitative data at best represent the unique perspectives of the study participants and contexts of analyses. Although we employed rigour in our qualitative approaches (including member checks, triangulation, and respondent validation) at best our findings are constrained by the limitations inherent in qualitative research. Moreover, our quantitative data were obtained through observational methods without comparison groups or randomization which affects the internal validity of our findings. In addition, the outcome of each quantitative study was constrained by the variables obtained during data collection, affecting external validity. Nonetheless, the use of multiple data sources derived from both qualitative and quantitative sources is a strength of the critical multiplism approach [55]. Future studies with more rigorous designs should be employed to examine specific features of smoke-free policies. For example, the use of comparator jurisdictions in our study design may have strengthened the transferability of our study findings.

## Conclusions

In this study, we explored the introduction of a smoke-free bylaw in one municipality. We learned that the bylaw was enthusiastically supported by both City and Park Board councillors and introduced with little opposition or debate. Yet our set of studies suggests that the ban on smoking in the parks and at the beaches in Vancouver has had mixed results to date. With a large inventory of parks and beaches and a small staff of enforcement officers, there is significant reliance on passive enforcement, and even when enforcement officers are engaged, a number of factors determine whether they will insist upon compliance in a given setting or circumstance. Our field observations suggest that there are some park and beach areas in which smoking continues, particularly in lower-income areas of the city, and that some populations of smokers are less likely to be challenged for non-compliance. Accordingly, both direct and SHS exposure may be differentially greater in some settings than others throughout the city’s parks and beaches.

The introduction of the smoke-free bylaw changed the role of the city’s Park Rangers. In choosing to make the Rangers responsible for bylaw enforcement, greater attention should have been given at the outset to developing their skills in handling the wide range of people they might encounter. In addition, given the numerous locations and vast network of parks and beaches, decision makers should have allocated sufficient resources to hire an appropriate number of officers and planned for meeting the needs of more disadvantaged smokers.

As public and population health researchers, we are committed to ensuring that population health interventions meet the aims of both tobacco control *and* health equity [58]. This means ensuring that the developers and implementers of outdoor smoke-free bylaws consider how the bylaw may affect various populations of smokers and develop strategies to improve the way that the bylaw is managed to ensure that it does not inadvertently contribute to greater health inequities. This might mean providing better training to enforcement officers in dealing with non-compliant park and beach users. It might also mean reducing the fine level so that it is less onerous for low-income violators, and providing greater supports for smoking reduction and cessation in the community. To increase the likelihood that lower-income smokers could enjoy the benefits of smoke-free parks, enforcement should be consistent across settings, and resources for enforcement should be adequate. Finally, proponents of tobacco control should join with the broader public and population health community in advocating for action on the determinants of health; adequate housing, income, education, and social equity are likely to contribute to reducing tobacco use in the first place and hence the demand for smoking in the park or at the beach.
